# Surface-Modified Superparamagnetic Iron Oxide Nanoparticles (SPIONs) in a New Perspective for Prostate Cancer Therapy

**DOI:** 10.2147/NSA.S526094

**Published:** 2026-01-14

**Authors:** Karolina Karnas-Janota, Czesław Kapusta, Janusz Przewoźnik, Martyna Kowalczyk, Anna Karewicz, Joanna Dulińska-Litewka

**Affiliations:** 1 Medical Biochemistry, Jagiellonian University Medical College, Cracow, Poland; 2 Department of Chemistry, Jagiellonian University, Cracow, Poland; 3 Faculty of Physics and Applied Computer Science, AGH University of Science and Technology, Cracow, Poland

**Keywords:** EMT, prostate cancer, signaling pathway, superparamagnetic iron oxide nanoparticles

## Abstract

**Purpose:**

Prostate cancer circulating tumor cells (PCTCs) are often found in the blood of patients suffering from metastatic prostate cancer and they are responsible for contributing to metastatic progression. Superparamagnetic Iron Oxide Nanoparticles (SPIONs) have been widely studied in the context of biomedical applications. Recently, circulating tumor cells (CTCs) capture and neutralization, as well as magnetically assisted drug delivery, have attracted much attention of researchers. Our studies are focused on the impact of the SPIONs stabilized with both cationic (CCh) and anionic (ACh) derivatives of chitosan on the model prostate cancer cell lines differing in phenotype and malignancy.

**Patients and Methods:**

In the research conducted, SPION/CCh and SPION/ACh particles were prepared, their colloidal stability and magnetic properties were examined using dynamic light scattering (DLS) technique, fluorescence spectroscopy, Mössbauer spectroscopy and magnetometry, and their impact on the properties of prostate cells (PC-3, LNCaP and DU 145) with various degrees of malignancy (normal and cancer) was determined in correlation with proteins of the cell signaling pathways involved in the epithelial-mesenchymal transition (EMT).

**Results:**

The SPION nanoparticles obtained were spherical, colloidally stable, and exhibited excellent magnetic properties. They showed an inhibiting effect on the migration of prostate cancer cells studied. Additionally, they slightly changed the expression of EMT pathway proteins, with an observed increase in E-cadherin which indicates, for the first time, a protective effect of SPIONs. The optical and confocal microscopy results obtained for the three cell lines studied indicated that the nanoparticles get internalized and also adsorbed on their surface, which is a desirable novel effect for their potential use as drug carriers in cancer therapy.

**Conclusion:**

The results obtained allow us to be the first to conclude that our SPION particles in non-toxic concentrations can be used as carriers of active substances for prostate cancer cells.

## Introduction

Prostate cancer is the second most commonly diagnosed malignancy in men and the fifth leading cause of cancer-related deaths worldwide.^1^ Although advances in early detection and localized treatment have improved patient survival, the management of advanced and metastatic disease remains challenging.^2^ Resistance to androgen deprivation therapy and standard chemotherapeutics highlights the urgent need for innovative therapeutic strategies that combine targeted delivery with minimal systemic toxicity.^3^

The rapid development of nanotechnology nowadays results in the widespread use of nanomaterials in almost all areas of life, such as electronics, cosmetics or medicine.^4–7^ Nanomedicine, an interdisciplinary field applying nanotechnology to medicine, offers promising approaches to cancer diagnosis and treatment. Ongoing research includes cutting-edge diagnostic therapies, imaging methods and treatments for various diseases.^8–10^ Due to the fact that neoplastic diseases are one of the most serious problems of civilization and that some of the current therapies are less effective than we would like and show adverse side effects for patients, there is a lot of literature on this group of diseases.^11–13^ Among its key areas of development are drug delivery systems (DDSs), which can be engineered in various sizes, shapes, and compositions to transport therapeutic agents directly to tumor sites in a controlled manner.^14–16^ Such targeted delivery has the potential to enhance treatment efficacy while reducing the administered drug dose and minimizing side effects compared with conventional chemotherapy.^17–19^ However, introducing nanocarriers into the human body can provoke adverse responses, including organ accumulation, disruption of biological barriers, oxidative stress, or DNA damage.^20–22^ These risks necessitate careful design and thorough biological evaluation of any nanosystem before clinical application.

Superparamagnetic iron oxide nanoparticles (SPIONs) have emerged as particularly promising multifunctional platforms owing to their magnetic responsiveness, biocompatibility, high surface area-to-volume ratio, and tunable surface chemistry.^23–25^ Clinically approved as MRI contrast agents, SPIONs are also being actively explored for magnetic hyperthermia, targeted drug delivery, and real-time therapy monitoring.^26–29^ Compared with other nanocarriers - such as liposomes, dendrimers, or gold nanoparticles - SPIONs offer the unique advantage of magnetic field-guided localization, enabling site-specific delivery and imaging enhancement.^30^,^31^ They also tend to exhibit lower systemic toxicity and are more efficiently cleared than many other inorganic nanoparticles. The biological performance of SPIONs depends strongly on their physicochemical properties. Particle size affects circulation time, biodistribution, and tumor accumulation via the enhanced permeability and retention (EPR) effect, with diameters in the 10–100 nm range generally considered optimal for tumor targeting.^32^,^33^ Shape can also influence uptake rates, with spherical particles often internalized more efficiently than rod-like morphologies.^34^ Surface chemistry plays a critical role in colloidal stability, biodistribution, cellular uptake, and toxicity. Cellular uptake of SPIONs, typically occurs via receptor-mediated endocytosis,^35^ influenced by surface charge, ligand density, and particle size. Uptake can begin within minutes and reach substantial intracellular concentrations within a few hours.^34^,^35^ Once internalized, SPIONs are trafficked to endosomes and lysosomes, where they can be engineered to release therapeutic cargo or serve as magnetic hyperthermia agents.^36^ Notably, positively charged SPIONs often show higher internalization rates, but this can be accompanied by increased cytotoxicity, whereas anionic coatings may favor biocompatibility but with slower uptake kinetics.

Coating constitutes the indispensable component of SPIONs used in biomedical applications. It should provide them with colloidal stability, increase their biocompatibility, and prevent opsonization and interactions with plasma proteins increasing their circulation in the bloodstream. Polymers, and polysaccharides in particular, are among the most frequently used SPION coatings, as they fit well in the above listed requirements. Those most often used are alginate, chitosan, dextran, heparin, starch and hyaluronic acid.^37^ While most of the abovementioned polysaccharides are negatively charged or neutral, chitosan is cationic and thus plays a special role in many biomedical applications. It has been widely used in drug delivery,^38^ gene delivery,^39^ self-healing injectable hydrogels,^40^ wound dressings^41^ or antibacterial materials.^42^ Chitosan is a copolymer of a 2-amino-2-deoxy-D-glucose and 2-acetamido-2-deoxy-D-glucose units linked with the (1–4) bonds. The presence of the hydroxyl and amino groups allows for the effective coating of iron oxides. It also facilitates crossing cell membranes and other biological membranes eg due to the ability of chitosan to open tight junctions of epithelial cells.^43^ The main limitation of chitosan is the ability to dissolve in acidic solutions, but not in water or physiological media. Its trimethylammonium salt, however, due to the presence of stable cation, is well soluble in aqueous media in a broad pH range, providing SPIONs with high colloidal stability and hydrophilicity. To study the cellular interactions with SPIONs having the coating based on the same polysaccharide backbone, but opposite charge, anionic derivative of chitosan can be used.

We have therefore synthesized and characterized SPIONs stabilized by a chitosan-based coating and having a positive or negative surface charge and conducted a series of biological studies to understand their effects on model prostate cancer cell lines differing in phenotype and expression of proteins involved in the EMT transition, a key regulator of cell invasion and metastasis in cancer.

## Materials and Methods

### Materials

Cationic and anionic derivatives of chitosan were synthesized according to previously obtained procedure,^44^ iron (II) chloride tetrahydrate (puriss. p.a., ≥ 98.5%, Sigma-Aldrich), iron (III) chloride hexahydrate (puriss. p.a., ≥ 98.5%, Sigma-Aldrich), ammonia (25% solution, puriss. p.a., POCh), 1,10-phenanthroline monohydrate (p.a., POCH S.A)., L-ascorbic acid (99%, Sigma Aldrich), formaldehyde solution (for molecular biology, 36.5–38% in H_2_O, Sigma Aldrich), Prussian blue soluble (for microscopy, Sigma Aldrich), Safranin O (Dye content ≥85%, Sigma Aldrich), fluorescein isothiocyanate (FITC, BioReagent, Sigma-Aldrich), PC-3 cell line (ATCC^®^), DU 145 cell line (ATCC^®^), LNCaP clone FGC cell line (ATCC^®^), RPMI 1640 culture medium (Gibco, ThermoFisher Scientific), Fetal Bovine Serum (FBS) (Sigma-Aldrich), Thiazolyl Blue Tetrazolium Bromide (MTT, powder, BioReagent, Sigma-Aldrich), DAPI (for nucleic acid staining, Sigma-Aldrich), rabbit anti-E-cadherin antibody, rabbit anti-N-cadherin antibody, rabbit anti-P-cadherin antibody, rabbit anti-PTEN antibody, rabbit anti-⍺-E-catenin antibody, rabbit anti-β-catenin antibody, rabbit anti-ZEB-1 antibody, rabbit anti-PCNA antibody, rabbit anti-Vimentin antibody, rabbit anti-HSP90 antibody, rabbit anti-HSP60 antibody, rabbit anti-Akt antibody, rabbit anti-Bad antibody, rabbit anti-BIM antibody, mouse anti-GSK antibody, rabbit anti-Bcl-2 antibody, rabbit anti-AR antibody, rabbit anti-β-actin antibody, anti-rabbit IgG HRP-conjugated antibody were all obtained from Cell Signaling Technology and were used as received. All compounds are >95% pure by HPLC.

### Synthesis of Magnetic Nanoparticles

**Figure 1:**
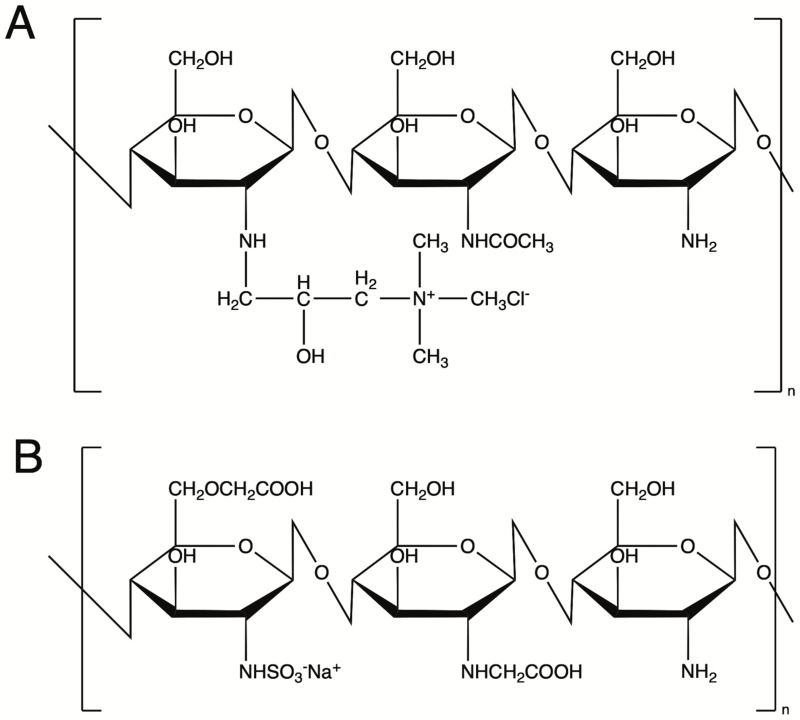
Chemical structures of the chitosan derivatives: CCh (**A**), ACh (**B**).

Cationic superparamagnetic iron oxide nanoparticles (SPION/CCh) were synthesized according to the protocol developed by Zapotoczny et al.^45^ The SPION/CCh nanoparticles were prepared via the co-precipitation of iron (II) and iron (III) oxides in the presence of a cationic chitosan derivative. The cationic chitosan (CCh) was obtained by modifying the amino groups of chitosan using glycidyltrimethylammonium chloride.^44^ The chemical structure of CCh is illustrated in Figure 1A.

Specifically, the amount of 162.2 mg of ferric chloride hexahydrate (FeCl_3_·H_2_O) and 59.6 mg of ferrous chloride tetrahydrate (FeCl_2_·H_2_O) were added to 50 mL of a 3 g/L solution of CCh, dissolved in 0.1 M sodium chloride. To ensure anaerobic conditions, the reaction mixture was purged with argon gas. Additionally, the flask was placed in an ice bath, and the solution was subjected to pulsed sonication to minimize nanoparticle aggregation during nucleation. Subsequently, 5 M aqueous ammonia was added dropwise under continuous stirring. After 30 minutes, the resulting colloidal suspension was purified using magnetic chromatography to remove excess of ammonia and non-magnetic impurities.

To obtain negatively charged nanoparticles (SPION/ACh), the positively charged SPION/CCh were coated with an anionic chitosan derivative containing carboxylic functional groups (ACh); as illustrated in Figure 1B. The coating procedure followed the methodology published by Szpak et al.^45^,^46^ An aqueous solution of ACh (1 g/L in a 0.2 M sodium chloride) was mixed with the SPION/CCh suspension at a 1:1 volume ratio. The mixture was then sealed and subjected to continuous sonication for 10 minutes. Finally, the coated nanoparticles were purified again via magnetic chromatography.

### Determination of Iron Concentration of the SPION Suspensions

The iron content in the SPION samples was quantified using colorimetric assay.^47^ To initiate the analysis, 0.1 mL of 3 M hydrochloric acid was added to 0.5 mL aliquots of both SPION/CCh and SPION/ACh suspensions. The mixtures were incubated overnight to ensure the complete dissolution of the iron oxide cores. Following the 24-hour incubation, 4.2 mg of ascorbic acid was introduced to each sample. The solutions were then left to stand overnight to allow for the full reduction of Fe (III) to Fe (II). Subsequently, the samples were diluted 100-fold and 2 mL of each diluted solution was combined with 1 mL of 0.1 M 1,10- phenanthroline. The mixtures were incubated for 1 hour in the dark to prevent photodegradation of the complex. UV-Vis absorbance spectra were recorded using HP 8452A spectrophotometer and the absorbance at 512 nm (corresponding to the Fe (II)-phenanthroline complex) was measured. The concentration of iron in the SPION suspensions was calculated based on the previously established calibration curve.

### Physicochemical and Magnetic Properties of the Nanoparticles

The average hydrodynamic diameter and colloidal stability of SPION/CCh and SPION/ACh nanoparticles were assessed using dynamic light scattering (DLS) and electrophoretic light scattering (ELS) techniques. Measurements were performed with a Zetasizer Nano ZS (Malvern Instruments), and the data were acquired and processed using Malvern Zetasizer Nano software version 3.30. Structural characterization was conducted via X-ray diffraction (XRD) using a Siemens D5000 diffractometer operating at room temperature. Fourier-Transform Infrared (FTIR)^47^,^48^ spectra were obtained with a Nicolet iS10 FTIR spectrometer equipped with an attenuated total reflectance (ATR) accessory (Thermo Fisher Scientific), and the spectral data were analyzed using OMNIC FTIR software. Mössbauer spectroscopy on the ^57^Fe isotope (stable, 2% of natural abundance) was employed to investigate the magnetic dynamics of the nanoparticles. The measurements were carried out in transmission mode at room temperature using a constant acceleration spectrometer manufactured by “Elektronika Jądrowa” (Kraków, Poland). The source consisted of 10 mCi ^57^Co embedded in a rhodium matrix. Samples were prepared by lyophilizing 2 mL of each colloidal suspension. Magnetic properties were further characterized using a Vibrating Sample Magnetometer module of the Quantum Design Physical Property Measurement System (PPMS). Measurements were performed on freeze-dried samples of both SPION/CCh and SPION/ACh in the temperature range from 300 K down to 4 K under Zero Field Cooling (ZFC) and Field Cooling (FC) conditions. Magnetization hysteresis loops were also recorded within a magnetic field range from −90 to 90 kOe. All magnetic data were processed and analyzed using OriginPro 2022 software.

### Nanoparticles’ Stability in Cell Culture Conditions

To assess the colloidal stability of SPION/CCh and SPION/ACh nanoparticles under conditions simulating a cell culture environment, a turbidimetric method was employed. Nanoparticle suspensions at a concentration of 10 µg/mL were incubated in RPMI-1640 culture medium (without the addition of fetal bovine serum, FBS) under two temperature conditions: room temperature (~22 °C) and 37 °C. In parallel, stability tests were also conducted in deionized water and phosphate-buffered saline (PBS). The samples were collected at predetermined time points: 0, 1, 2, 3, 4, 5, 6, 8, 24 and 48 hours. At each interval, the optical density (OD) of the suspension was measured at wavelength of 700 nm using a UviLine SI 7000 spectrophotometer (SI Analytics). All measurements were conducted at room temperature using quartz cuvettes with a 1 cm optical path length. Changes in optical density over time were analyzed to monitor nanoparticle aggregation and sedimentation behavior, with all data processing performed using OriginPro 2025 software.

### Labelling SPIONs with Fluorescent Dye for Microscopic Imaging

Fluorescent labeling of SPION/CCh and SPION/ACh nanoparticles was performed using fluorescein isothiocyanate (FITC). Specifically, 1 mL of the FITC solution in methanol (2 mg/mL) was added to 4 mL of each nanoparticle suspension. The mixtures were incubated for 6 hours at room temperature in the absence of light to facilitate covalent attachment of the dye. Following incubation, the samples were transferred into dialysis tubing (cellulose membrane, molecular weight cut-off: 4 kDa, Sigma Aldrich) and dialyzed against a 1:1 (v/v) mixture of deionized water and ethanol. Dialysis was continued until all unbound FITC was removed.

### Cell Cultures

The study was conducted using human prostate normal cell line: RWPE-1 (immortalized epithelial cell line, derived from normal adult prostate tissue) and four human prostate cancer cell lines: LNCaP (androgen dependent prostate cancer from bone metastases), PC-3 (androgen independent prostate cancer from bone metastases), DU 145 (androgen independent prostate cancer from brain metastases) and 22Rv1 (androgen-dependent, derived from a human prostate carcinoma xenograft). Normal prostate cell line was cultured in Keratinocyte Serum-Free Medium (K-SFM) supplemented with bovine pituitary extract (BPE) and epidermal Growth Factor (EGF). All cancer cell lines were cultured in RPMI-1640 medium supplemented with 10% (v/v) fetal bovine serum (FBS). Cells were maintained under standard conditions in a humidified incubator at 37 °C with 5% CO_2_ and 90% relative humidity. To ensure experimental reliability, all cell lines were routinely tested for mycoplasma contamination using polymerase chain reaction (PCR)-based detection.

### Cytotoxicity Assay

To evaluate the cytotoxicity of SPION/CCh and SPION/ACh nanoparticles, RWPE-1, PC-3, LNCaP DU 145 and 22Rv1 cells were seeded into 96-well culture plates at a density of 4,500 cells per well in 200 µL of culture media: supplemented K-SFM medium (RWPE-1) and complete RPMI-1640 medium supplemented with 10% (v/v) fetal bovine serum. Cells were incubated under standard conditions (37°C, 5% CO_2_, 90% humidity) to allow adherence for 24 hours (48 hours for LNCaP cells) to allow them to adhere to the well surface. After the adhesion period, the culture medium was replaced with 100 µL of a fresh one containing SPION nanoparticles at concentrations ranging from 1 to 100 µg/mL. The cells were further incubated for 24 hours and 48 hours under standard culture conditions. Cell viability was assessed using the MTT assay. Following incubation with the nanoparticles, 5 µL of MTT reagent (5 mg/mL in PBS) was added to each well. The plates were incubated for additional for 3 hours at 37°C. Subsequently,50 μL of lysis buffer (10% SDS in 0.01 M HCl) was added to each well to solubilize the formed formazan crystals and the plates were incubated overnight in the dark at 37°C. Absorbance was measured at 570 nm using ELISA microplate reader (Synergy HT, BIO-TEK, USA). The obtained data were analyzed using KC4 Software. Absorbance values were normalized to untreated control wells (cells cultured without nanoparticles), and cell viability was expressed as a percentage of the control. Each condition was tested in technical triplicates.

### Confocal Microscopy

PC-3, DU 145 and LNCaP prostate cancer cells were seeded onto sterile glass coverslips placed in a 6-well plate at a density of 8,000 cells per well. Cells were incubated for 24 hours (48 hours for LNCaP) under standard conditions (37 °C, 5% CO_2_, 90% humidity) to allow for adhesion. Subsequently, the culture medium was replaced with fresh medium containing fluorescently labelled SPIONs (5 µL/mL) and the cells were incubated for additional 24 hours. Following incubation, the cells were washed twice with sterile PBS and fixed with 4% formaldehyde for 20 minutes at room temperature. Coverslips were mounted onto microscopic slides and secured. Fluorescence imaging was performed using a Nikon Ti-E inverted microscope (Precoptic) equipped with the Nikon A1 confocal system, 405 and 488 nm lasers lines, DAPI and FITC filter sets, and a 100x oil-immersion objective. Image acquisition and analysis were conducted using the NIS-Elements software suite. To assess the time-dependent interaction of nanoparticles with cells, additional experiments were performed using fluorescently labelled SPION/CCh nanoparticles at a concentration of 10 µg/mL. Two cell lines: PC-3 and DU 145 were incubated with the nanoparticles for 1, 5 and 24 hours. Slide preparation and imaging were conducted as described above.

### Prussian Blue Staining

To visualize iron content within the magnetic cores of SPIONs, Prussian blue staining was performed, with safranin O used as a counterstain. PC-3 and DU 145 cells were seeded, and cultured on glass coverslips as described previously. Cells were incubated with non-labeled (unstained) SPIONs, fixed with 4% formaldehyde, and subsequently washed thoroughly wit PBS. For iron staining, 300 µL of a freshly prepared solution containing 2% (w/v) potassium ferrocyanide (Prussian Blue) in 2% (v/v) hydrochloric acid was added to each well. The plates were incubated in dark at room temperature for 30 minutes. Following staining, cells were washed three times with PBS to remove excess reagent. Counterstaining was performed by incubating the samples with 300 µL of 0.1% safranin O solution for 30 minutes at room temperature. The stained cells were again washed with PBS and mounted for microscopic analysis. Visualization of iron deposits was carried out using a Nikon Ti-E inverted microscope equipped with a 10x objective. Microscopic images were acquired to assess the intracellular localization and distribution of SPION-derived iron.

### Scratch Assay

A scratch (wound healing) assay was conducted to assess the impact of SPIONs on the migratory potential of prostate normal and cancer cells. RWPE-1, PC-3, DU 145, LNCaP and 22Rv1 cells were seeded in 24-well plates at a density of 35,000 cells per well and incubated under standard conditions until a confluent monolayer was formed. At confluence, the medium was gently aspirated and replaced with fresh medium, either without nanoparticles (control) or supplemented with SPION/CCh or SPION/ACh at iron equivalent to 1 µg/mL, 5 µg/mL or 10 µg/mL. A linear scratch was then introduced into the cell monolayer using a sterile 200 µL pipette tip. Brightfield images of the scratch area were captured at 0, 24, and 48 hours post-wounding using an inverted light microscope equipped with a digital camera. The wound closure was quantified by measuring the scratch area at each time point using ImageJ software (NIH, USA). The percentage of wound closure was calculated relative to the initial wound area. Quantitative data were statistically analyzed and plotted using ImageJ and OriginPro 2025 software. Experiments were performed in triplicate.

### Western Blot Analysis

To evaluate the impact of SPIONs on key cellular processes such as apoptosis, proliferation, and metastasis, Western blot analysis of protein expression was performed across one prostate normal cell line (RWPE-1) and four prostate cancer cell lines: PC-3, DU 145, LNCaP and 22Rv1. Cells were seeded in a 6-well culture plates at a density of 950,000 cells per well and incubated in culture media for 24 hours (48 hours for LNCaP) to allow for adhesion. After incubation, the media was replaced with fresh media SPION/CCh or SPION/ACh at iron-equivalent concentration of 0, 1, 5 and 10 µg/mL. Treated cells were incubated for additional 24 or 48 hours. Following incubation, cells were lysed using a standard solubilization buffer. Lysates were subjected to thermal denaturation at 95 °C for 5 minutes, homogenized using pulsed sonication (10 seconds), and subsequently clarified by centrifugation. Total protein concentration in each sample was quantified the QubitTM fluorometric assay following the manufacturer’s protocol. Protein expression was analyzed by SDS-PAGE and immunoblotting. For each condition, 30 µg of total protein was loaded onto 10% Tris-Glycine gel and separated by electrophoresis. Proteins were transferred onto PVDF membranes, which were then blocked for 1 hour at room temperature with 5% (w/v) non-fat dry milk in TBS/T (Tris-buffered saline with 0.1% Tween-20). Membranes were incubated overnight at 4°C with the appropriate primary antibodies [N-cadherin, E-cadherin, P-cadherin, PTEN, AKT, β-catenin, α-E-catenin, AR, HSP-60, PCNA, Caspase-9, BIM, BCL-2, BAD, BIM and housekeeping protein β-actin – LabJot – Cell signaling Technology]. After washing, membranes were incubated with horseradish peroxidase (HRP)-conjugated secondary antibodies. Protein bands were visualized using a Bio-Rad ChemiDoc^TM^ imaging system, and densitometric analysis was performed using Image Lab software. All samples were analyzed in triplicate. Relative protein expression levels were normalized to β-actin as a loading control. Experiments were performed in triplicate.

### Statistical Analysis

All statistical analyses were performed using GraphPad Prism software (version 8.0.1, GraphPad Software, San Diego, CA, USA). For MTT assay and scratch assay results, three-way analysis of variance (ANOVA) was performed, followed by Tukey’s multiple comparisons test to assess differences between experimental groups. Each experiment was conducted in triplicate, and results are expressed as the mean ± standard deviation (SD). Statistical significance was set at p < 0.05, indicated as * (black for SPION/CCh and red for SPION/ACh). Comparisons between individual variables in Western blot analysis were performed using two-way ANOVA. Dunnett’s multiple comparisons test was used to determine which values differed significantly from the respective controls. Statistical significance was set at p < 0.05, with p < 0.05 indicated as *and p < 0.01 indicated as **.

## Results

### Synthesis of SPIONs

SPION/CCh were obtained using a popular precipitation method. Iron (II) and iron (III) salts were precipitated using ammonia in the presence of the cationic derivative of chitosan, synthetized previously using glycidyltrimethylammonium chloride (GTMAC) as a reagent. To prevent the oxidation of iron (II) during precipitation the process was performed under the constant flow of argon. To provide sufficiently strong mixing in order to prevent early aggregation of nanoparticles, before their coating is fully formed, the mixture was sonicated during the precipitation process. SPION/ACh were obtained based on SPION/CCh, by the incubation of the cationic nanoparticles in the solution of ACh.

### Physicochemical Properties of SPION/CCh and SPION/ACh

To characterize the synthetized nanoparticles, their average hydrodynamic diameter, polydispersity index (PdI) and zeta potential values were measured using DLS and ELS techniques. The results, along with the average iron concentrations determined via spectrophotometric analysis, are summarized in Table 1. The SPION/ACh sample exhibited a negative zeta potential, confirming the successful formation of a polymeric anionic layer on the surface of SPION/CCh. Comparable average particle sizes of SPION/CCh and SPION/ACh suggest that this additional layer is relatively thin. In both formulations, the low PdI value indicates a relatively uniform size distribution of the nanoparticles. Furthermore, the high absolute value of the zeta potential points to their good colloidal stability. The measured iron concentrations of SPION/CCh and SPION/ACh suspensions reveal a degree of nanoparticles loss during the surface modification and subsequent magnetic purification steps.

**Table 1:** Physicochemical Properties of the SPION/CCh and SPION/ACh Suspensions

Sample	Size [nm]	PdI	Zeta Potential [mV]	Iron Concentration [ug/mL]
SPION/CCh	153 ± 19	0.181 ± 0.011	43.7 ± 1.6	395.8 ± 2.6
SPION/ACh	248 ± 16	0.157 ± 0013	-(48.9 ± 1.2)	340.5 ± 12.2

**Figure 2:**
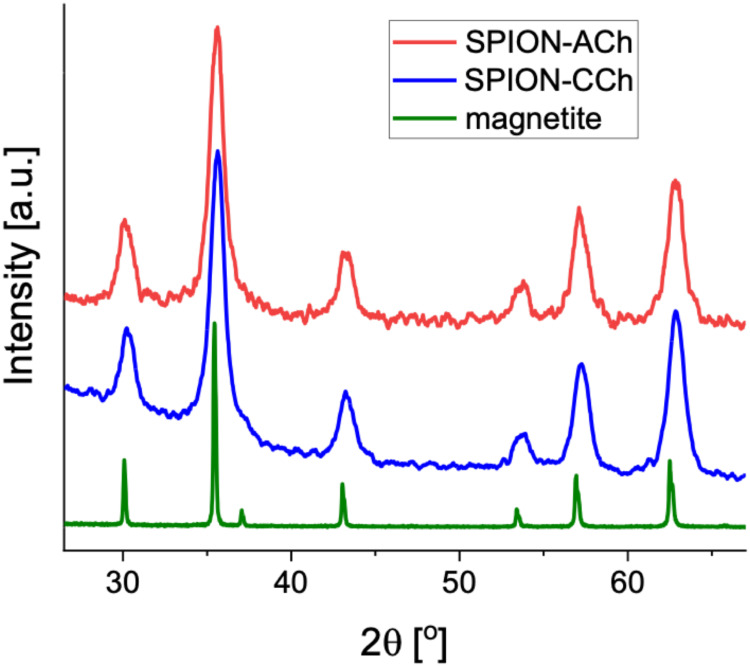
Diffractograms of SPION/CCh and SPION/ACh and the reference of microcrystalline magnetite.

X-ray diffraction (XRD) patterns of SPION/CCh and SPION/ACh (Figure 2) confirm that the magnetic cores are nanocrystalline and possess the inverse spinel structure characteristic of magnetite. Using the Scherrer equation to correlate peak broadening with crystallite size, the estimated average core diameter of approximately10 nm for both nanoparticle types was obtained. A slight shift of the diffraction peaks towards higher angles relative to standard magnetite suggests a reduction in lattice constants. This shift implies that the cores may consist of maghemite, an oxidized form of magnetite, characterized by the presence of cation vacancies and an increased average oxidation state of Fe approaching +3.

**Figure 3:**
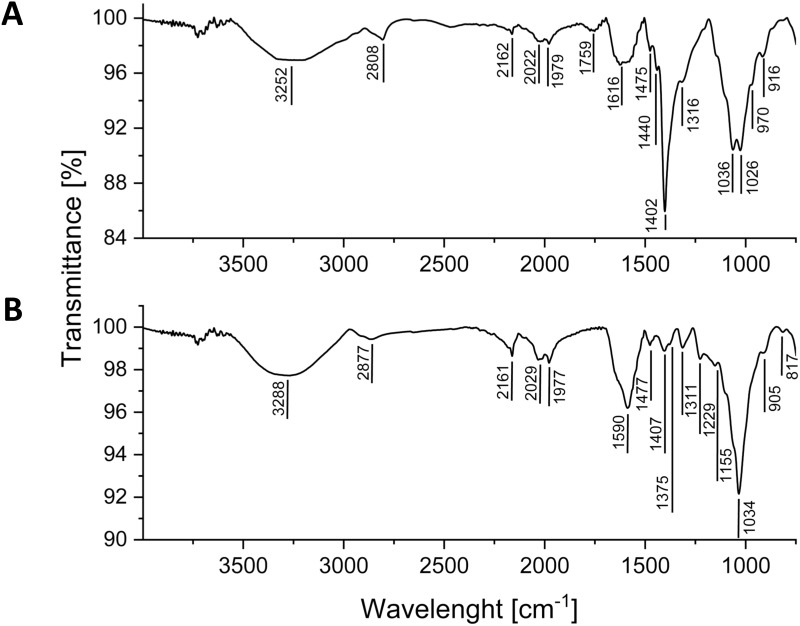
FTIR spectra of SPION/CCh (**A**) and SPION/ACh (**B**).

Reflectance Fourier Transform Infrared (ATR-FTIR) spectra (Figure 3) confirm the successful surface modification of SPION/CCh with ACh. Both SPION variants exhibited common bands characteristic for chitosan chain: a broad band centered at approximately 3250–3290 cm^−1^ corresponding to the O-H and N-H stretching vibrations and the peak at 2877 cm^−1^ corresponding to the C-H stretching vibrations. In the SPION/CCh spectrum, the band characteristic for C-H vibrations in the main polysaccharide chain is partially obscured by the band at 2808 cm^−1^ corresponding to the C-H stretching vibrations of methyl groups in the trimethylammonium substituent. The weak band at 1759 cm^−1^ in the spectrum of SPION/CCh can be attributed to amide I (C=O stretching vibrations) of the chitosan backbone and is not visible in the spectrum of SPION/ACh, possibly due to the differences in deacetylation degree of both derivatives. The bands at 1616 cm^−1^ (asymmetric deformations) and at 1402 cm^−1^ (symmetric CH deformations) in SPION/CCh spectrum corresponds to the trimethylammonium group, while the band at 1590 cm^−1^, more pronounced for SPION/ACh, is characteristic for deformation vibrations of primary amines in chitosan’s backbone. In the spectrum of SPION/ACh there are also bands characteristic for carboxylic group at 1407 cm^−1^ and 1375 cm^−1^ (CO stretching and OH deformation vibrations) and those corresponding to –SO_3_ group at 1229 cm^−1^ (asymmetric stretching) and 1155 cm^−1^ (symmetric stretching). The remaining bands visible in both spectra correspond to the vibrations of the pyranose unit of chitosan.

### Magnetic Characterization of SPIONs

**Figure 4:**
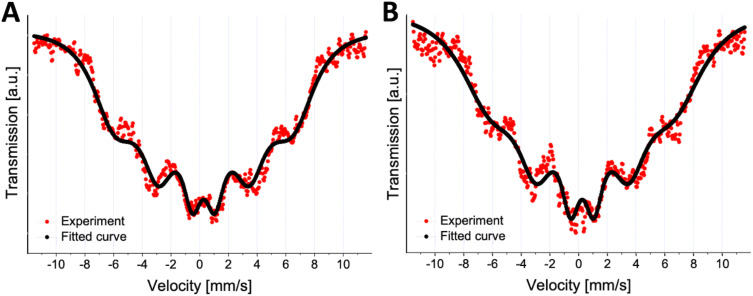
^57^Fe Mössbauer spectra of SPION/CCh (**A**) and SPION/ACh (**B**) at room temperature.

The Mössbauer spectra recorded at room temperature for dried nanoparticle materials (Figure 4) exhibit a relaxational profile, characterized by a sextet of strongly broadened overlapping lines. This spectral shape is indicative of superparamagnetic relaxation, reflecting thermally driven fluctuations of the magnetic moment of the nanoparticle as a whole. Spectral fitting based on the Tjon and Blume model,^49^ provided the values of fluctuation rates of 96(7) MHz and 62(4) MHz for the CCh and ACh SPIONs, respectively, confirming their superparamagnetic behavior.

**Figure 5:**
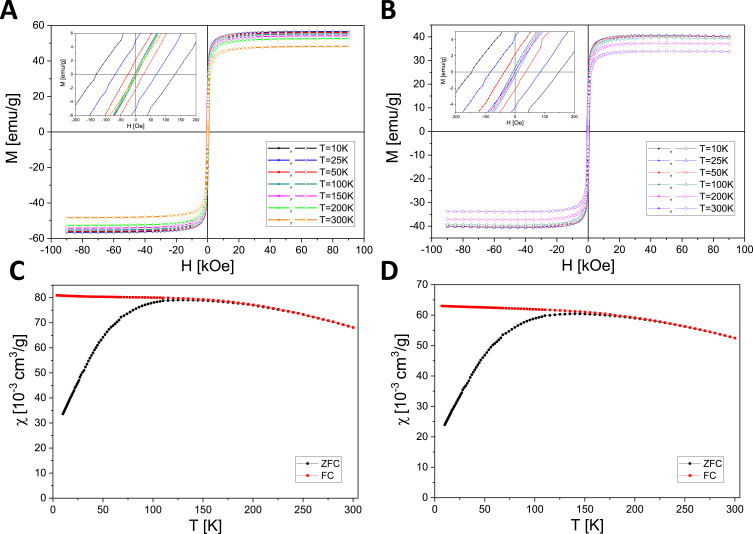
Magnetic field dependencies of the magnetization of SPION/CCh (**A**) and SPION/ACh (**B**) at selected temperatures. The insert shows a zoom at the origin. Temperature dependencies of the magnetic susceptibility of SPION/CCh (**C**) and SPION/ACh (**D**) taken in the ZFC mode and FC mode.

Complementary magnetometric measurements further support these findings. At low temperatures, both SPION/CCh and SPION/ACh nanoparticles exhibit ferromagnetic characteristics as evidenced by the presence of hysteresis loops (inset in Figure 5). However, their widths decrease to zero upon heating to 200 K and 300 K, consistent with the transition from a ferromagnetic to a superparamagnetic regime. Superparamagnetic relaxation behavior was also investigated by analyzing the magnetic susceptibility under zero-field-cooled (ZFC) and field-cooled (FC) conditions. ZFC measurements were performed by cooling the sample in the absence of a magnetic field, while FC measurements involved cooling in a constant applied field of 100 Oe. The blocking temperature (T_b_), defined as the peak of the ZFC curves (Figure 5) was found to be slightly below 200 K for both SPION/CCh and SPION/ACh. This observation aligns with the disappearance of coercivity at 200 K and confirms the transition to a superparamagnetic state above T_b_, where the magnetic moments of individual nanoparticles undergo rapid thermal fluctuations.

### Nanoparticles’ Stability in Cell Culture Conditions

**Figure 6:**
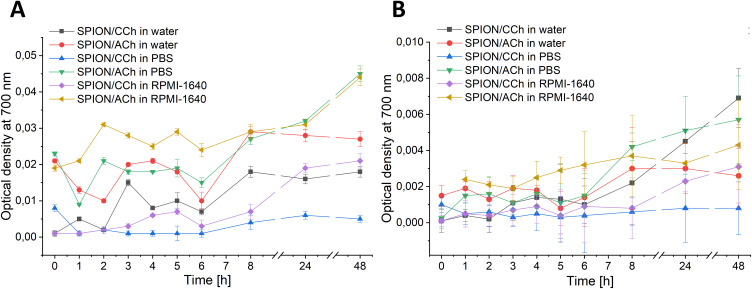
Optical density of the SPION/CCh and SPION/ACh suspensions in deionized water, PBS and RPMI-1640 medium measured at 700 nm at room temperature (**A**) and 37°C (**B**) after various incubation times.

Both SPION/CCh and SPION/ACh formulations demonstrated good colloidal stability under all tested conditions (water, PBS, and cell culture medium at room temperature and 37°C) over a 48h period. Although variations in optical density (OD) were detectable between different media and temperatures, absolute OD values remained low (typically in the 0.001–0.05 range for most conditions), indicating minimal nanoparticle aggregation and sedimentation (Figure 6). Notably, SPION/ACh generally exhibited slightly higher stability in aqueous environments, whereas SPION/CCh displayed more pronounced stability in PBS and culture medium. The observed differences were subtle rather than dramatic, suggesting that in subsequent biological experiments, nanoparticles remained largely well-dispersed. This minimizes the likelihood that aggregation or sedimentation significantly influenced cytotoxicity or migration assay results.

From a biological perspective, such stable dispersion is advantageous for nanoparticle–cell interactions, as it ensures uniform exposure of both cancerous and non-cancerous prostate cell lines to nanoparticles. This stability profile supports the potential application of both SPION formulations as drug delivery platforms or diagnostic agents, where maintaining consistent physicochemical properties in physiological environments is essential for reproducible therapeutic outcomes.

### Cytotoxicity of SPION/CCh and SPION/ACh

**Figure 7:**
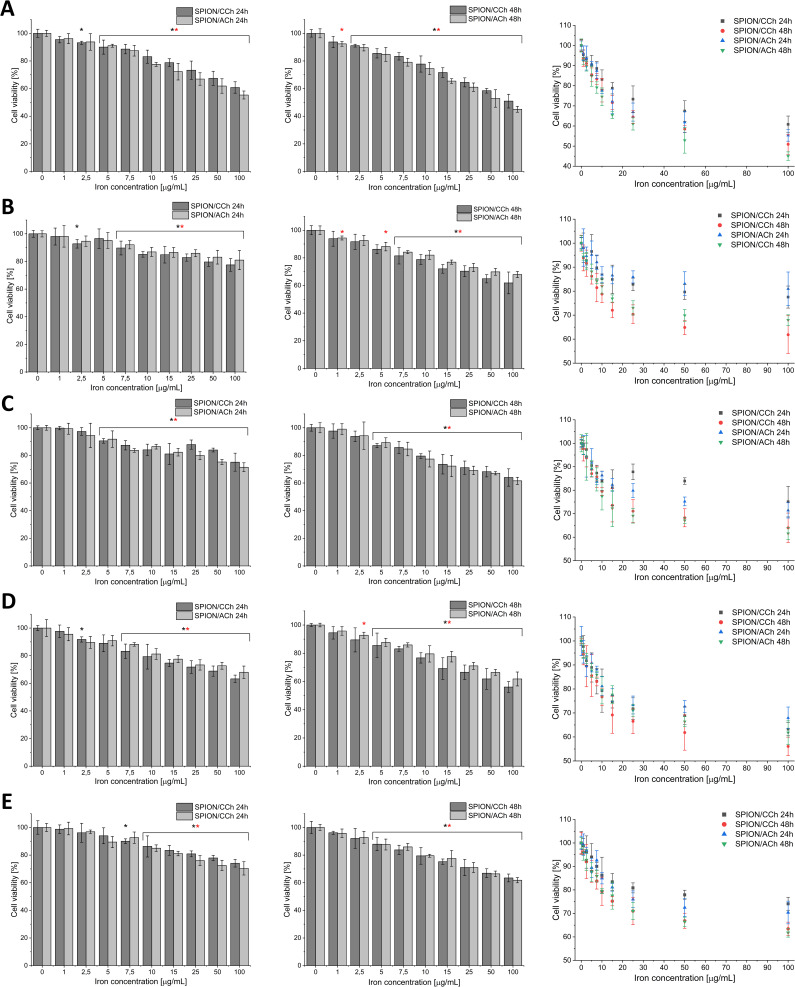
The results of MTT assay for RWPE-1 (**A**), PC-3 (**B**), DU 145 (**C**), LNCaP (**D**), and 22Rv1 (**E**) cells incubated with various concentrations of SPION/CCh and SPION/ACh for 24 and 48h. Statistical analysis was performed using one-way ANOVA: statistically significant are marked as * p > 0.05 deviations (black * for SPION/CCh and red * for SPION/ACh).

The assessment of nanomaterial toxicity is fundamental for subsequent biological studies and the potential application of these materials in clinical settings. In this study, the MTT assay was employed to evaluate the cytotoxicity of the synthesized nanoparticles, particularly in relation to their suitability for active substance delivering and capturing circulating prostate cancer cells. Additionally, the impact of the nanoparticles’ surface charge on their cytotoxicity was investigated. Figure 7 presents the results of the MTT assay for the prostate normal cell line (RWPE-1) and cancer cell lines PC-3, LNCaP, DU 145 and 22Rv-1 following 24-hour and 48-hour incubation with varying concentrations of nanoparticles. The MTT assay demonstrated a concentration- and time-dependent reduction in viability across all tested prostate cell lines following exposure to SPION/CCh and SPION/ACh (1–100 µg Fe/mL, 24 h and 48 h). In 22Rv1 cells, both nanoparticle types produced a gradual decline in viability, more evident after 48 h, with SPION/CCh showing slightly higher cytotoxicity. DU 145 and LNCaP cells were the most sensitive, displaying marked reductions at concentrations above 5 µg/mL, particularly after prolonged exposure; in these lines, SPION/CCh generally induced stronger effects than SPION/ACh. PC-3 cells showed a moderate viability decrease, with differences between coatings being cell-line dependent. RWPE-1 cells were relatively resistant, maintaining high viability except at the highest concentrations, where both nanoparticle types caused noticeable effects. Overall, the data indicate that nanoparticle cytotoxicity is influenced by surface charge and coating composition, with a tendency for greater selectivity toward malignant cells.

### Microscopic Imaging

**Figure 8:**
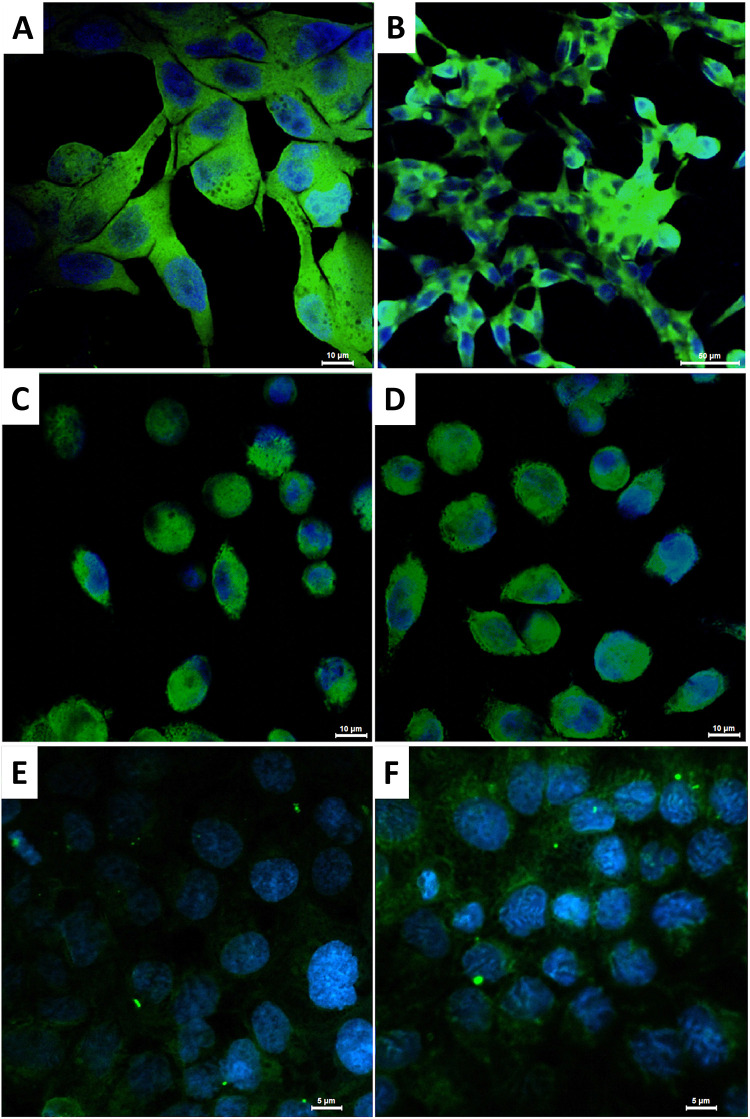
Images of LNCaP (**A** and **B**), PC-3 (**C** and **D**) and DU 145 (**E** and **F**) cells incubated with: SPION/ACh (**A, C** and **E**) and SPION/CCh (**B, D** and **F**); green fluorescence: FITC-labelled SPIONs, blue fluorescence: DAPI-stained cell nuclei; magnification: 100x (**A**) and 40x (**B**–**F**).

**Figure 9:**
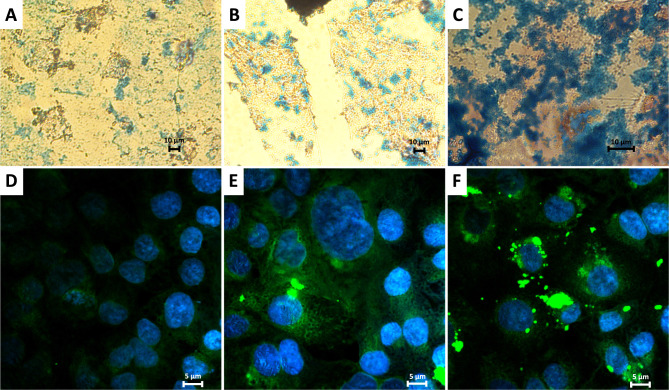
DU 145 cells incubated with SPION/CCh for: 1 h (**A** and **D**), 5 h (**B** and **E**) and 24 h (**C** and **F**). Images (**A**–**C**) were obtained with optical microscope (4x magnification) and show Prussian blue staining of iron oxide cores. Images (**D**–**F**) were obtained using confocal microscopy in a fluorescence mode (100x magnification) and show green florescence from the polymer coating labelled with FITC and blue fluorescence of the cell nuclei stained with DAPI.

**Figure 10:**
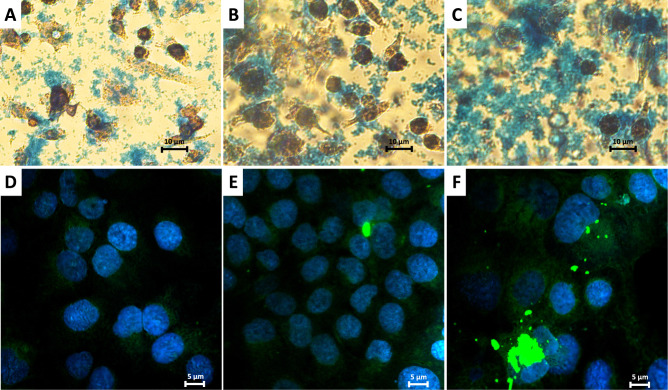
PC-3 cells incubated with SPION/CCh for: 1 h (**A** and **D**), 5 h (**B** and **E**) and 24 h (**C** and **F**). Images (**A**–**C**) were obtained with optical microscope (4x magnification) and show Prussian blue staining. Images (**D**–**F**) were obtained using confocal microscopy in fluorescence mode (100x magnification) and show green florescence from the polymer coating labelled with FITC and blue fluorescence of the cell nuclei stained with DAPI.

To investigate the influence of the surface charge of nanoparticles on their ability to enter and penetrate prostate cancer cells, as well as to visualize their intracellular distribution, microscopic imaging techniques were employed. The polymeric coating of the SPIONs was labelled with the fluorescent dye FITC, and imaging was performed using confocal microscopy. Complementary studies were conducted with optical microscopy, wherein the iron oxide cores of the nanoparticles were stained using Prussian blue to track their distribution within the cells. Furthermore, time-dependent studies were performed for the PC-3 and DU 145 cell lines to observe changes in the distribution of SPIONs over time. The resulting images are presented in Figures 8–10.

The images obtained for all three cell lines shown in Figure 7 confirmed that both SPION/CCh and SPION/ACh effectively penetrate the cells and are evenly distributed in the cytoplasm.^48^,^49^ Figures 8 and 9 provide a comparison of optical and confocal microscopy images illustrating the penetration of SPION/CCh after various incubation times. The green fluorescence signal corresponding to the FITC-labeled polymer coating, alongside the blue staining of iron oxide cores, reveals that over a 24-hour period, the number of nanoparticles inside the cells increases with time. After 24 hours, strongly fluorescent SPION aggregates become visible, which are too large to be internalized by the cells, and thus adsorb onto their surface. The trend is further corroborated by the optical microscope, where the amount of blue-stained iron increases with incubation time, demonstrating complementary results between two staining methods. The combined data from both microscopy techniques illustrate the dynamic process of nanoparticles internalization and their accumulation over time.

### Effect of SPIONs on Cell Migration

**Figure 11:**
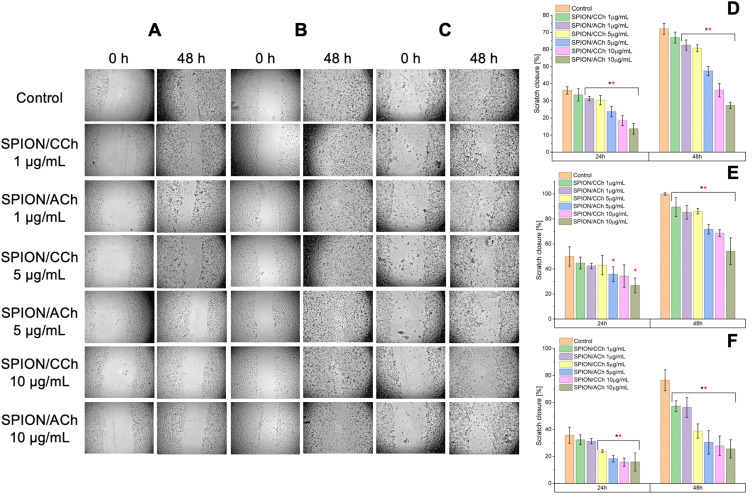
Scratch assay results obtained for RWPE-1 (**A** and **D**), PC-3 (**B** and **E**) and DU 145 (**C** and **F**) cell lines with graphs showing the change in the scratch surface area depending on the incubation time and the presence of SPION/CCh and SPION/ACh nanoparticles. Statistical analysis was performed using one-way ANOVA: statistically significant are marked as * p > 0.05 deviations (black * for SPION/CCh and red * for SPION/ACh).

**Figure 12:**
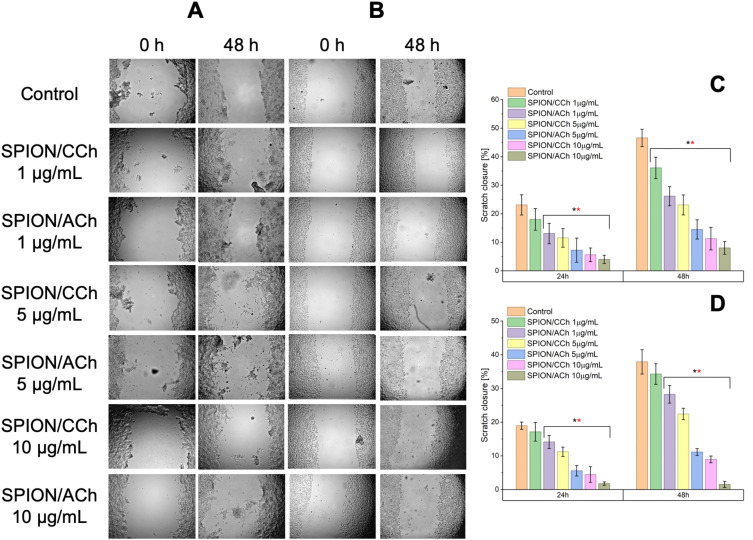
Scratch assay results obtained for LNCaP (**A** and **C**) and 22Rv1 (**B** and **D**) cell lines with graphs showing the change in the scratch surface area depending on the incubation time and the presence of SPION/CCh and SPION/ACh nanoparticles. Statistical analysis was performed using one-way ANOVA: statistically significant are marked as * p > 0.05 deviations (black * for SPION/CCh and red * for SPION/ACh).

The scratch assay was used to assess whether SPIONs influence the migration capacity of prostate cancer cells and normal prostate epithelial cells. Figures 11 and 12 present the results for all examined cell lines (PC-3, DU145, LNCaP, 22Rv1, and RWPE-1) immediately after SPION/CCh or SPION/ACh addition, and after 24 and 48 hours of incubation. Across all cell lines, a consistent trend was observed – higher nanoparticle concentrations led to reduced wound closure, particularly after longer incubation times. The magnitude of this effect depended on the concentration, nanoparticle surface charge (positive or negative), and the specific cell line.

In cancer cell lines LNCaP, 22Rv1, and DU145, migration decreased noticeably already at moderate concentrations (5–10 µg Fe/mL), with SPION/ACh generally exerting a stronger inhibitory effect than SPION/CCh. PC-3 cells required higher concentrations or longer exposure to reach similar levels of migration inhibition. Interestingly, the normal prostate epithelial cell line RWPE-1 displayed a trend similar to PC-3, with gradual inhibition of migration as concentration increased, and a marked reduction only at ≥5–10 µg Fe/mL after 48 h. At lower concentration (1µg Fe/mL), RWPE-1 cells maintained relatively high mobility, suggesting lower sensitivity in the early phase of exposure.

From a therapeutic standpoint, this is important – SPIONs can inhibit migration in both cancerous and normal prostate cells, but the threshold concentration differs. For RWPE-1, substantial inhibition occurs at concentrations that remain below the cytotoxicity threshold observed in the MTT assay, indicating that, with proper dose selection, it may be possible to limit the metastatic potential of cancer cells without significantly affecting healthy tissue. In the context of drug delivery and the capture of circulating tumor cells, these differences in sensitivity could be exploited by modifying nanoparticle surfaces and incorporating targeting ligands to direct them preferentially toward cancer cells, enhancing treatment efficacy while reducing off-target effects in healthy cells.

### Western Blot Analysis of Signal Pathways

**Figure 13:**
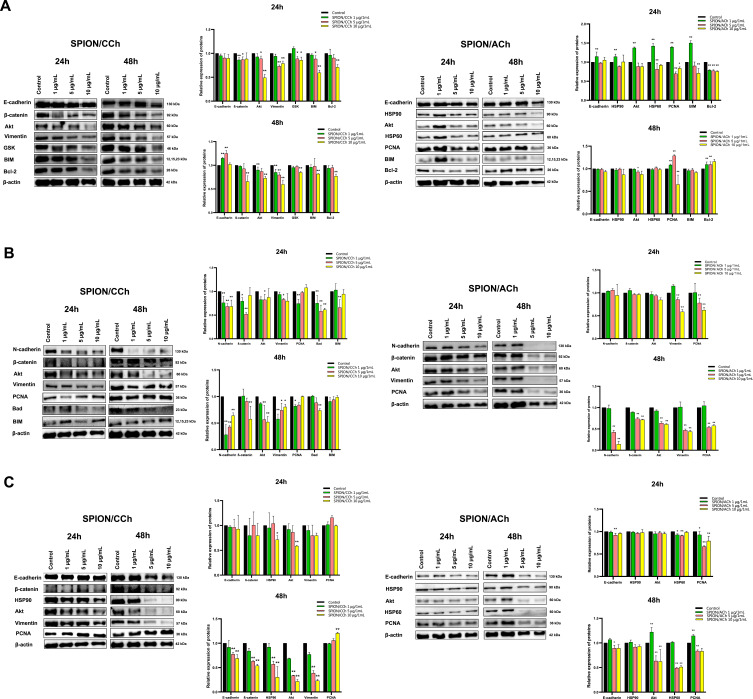
Western Blot analysis for RWPE-1 (**A**), PC-3 (**B**) and DU 145 (**C**) cell lines. Statistical analysis was performed using two-way ANOVA: statistically significant deviations are marked as * p > 0.05 and ** p > 0.01.

**Figure 14:**
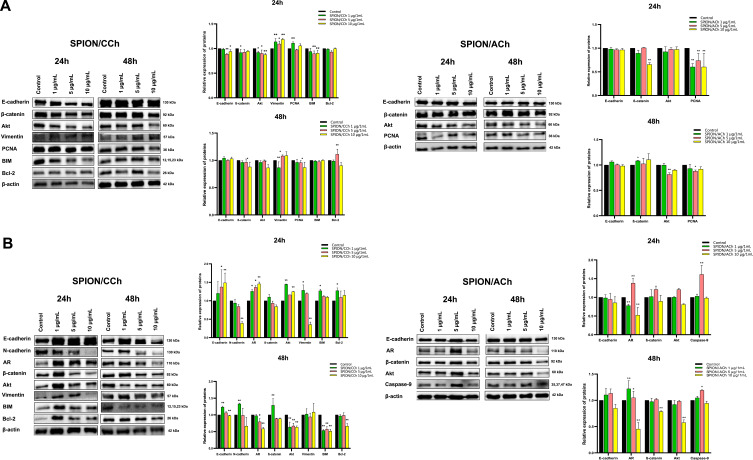
Western Blot analysis for LNCaP (**A**) and 22Rv1 (**B**) cell lines. Statistical analysis was performed using two-way ANOVA: statistically significant deviations are marked as * p > 0.05 and ** p > 0.01.

Knowing that SPION nanoparticles can penetrate prostate cancer cells and affect their migration capacity, their impact on signaling pathways was investigated. For this purpose, the level of expression of proteins involved in the epithelial-mesenchymal transition (EMT) in lysates of cells incubated with nanoparticles was examined (Figures 13 and 14).

For the PTEN protein [a tumor suppressor], we can observe a tendency that more intense bands were obtained for a lower concentration of the nanoparticles compared to a higher one. In addition, protein expression is higher after 48 h of incubation than in the case of 24 h, which is clearly visible for cells from the DU 145 line [data not shown]. In the case of beta-catenin, after treatment of SPION/CCh cells, we observe a tendency to decrease the expression of this protein, especially for the DU145 line, but also for the 22Rv1 line, where the changes are significant. The changes correspond to an increased concentration of iron. Only for the DU145 line, when the concentration of 1μg/mL is used, we see a significant increase in the expression of beta-catenin. No changes in the expression of this protein were observed for SPION/ACh. 24 h incubation of the nanoparticles with PC-3 caused a decrease in the intensity of the bands after treated with 1 and 5 µg/mL SPION/CCH, while after 48 h significant changes were also observed for the higher concentration. In addition, the use of a higher concentration of SPION/CCh causes a decrease in expression in a shorter time, and after a longer incubation, a decrease in expression is observed. In the case of the PC-3 line, after treatment of SPION/Ach cells, a significant, several-fold decrease in the expression of this protein was observed. Normal RWPE1 cells show a decrease in β-catenin expression after treatment with higher SPION/CCh concentrations, especially after 48 h of incubation, while no effect of SPION/ACh molecules on the expression of this protein was observed. Independent research indicates that, another catenin protein, α-E-catenin, was detected in the DU 145 line. After 24 h of incubation, a significant increase in band intensity was observed, which is additionally higher for SPION/ACh. After a longer time, a slight increase in expression was observed after treatment of SPION/CCh and SPION/ACh cells (the exception is a lower concentration of the latter). The analysis of cadherins started with the detection of N-cadherin in the PC-3 line. In general, insignificant changes in protein expression can be observed. Interestingly, it can be seen that for samples treated with SPION/CCh or SPION/ACh - resulted in a significant decrease in N-cadherin expression, especially after a longer incubation time. The decrease in the intensity of the bands occurs with the increase in the concentration of nanoparticles, and for anionic SPIONs, the trend is similar. For the 22Rv1 line, we observe significant decrease in N-cadherin expression after 24 hours of SPION/CCh treatment. The remaining cell lines do not express N-cadherin. In the case of another protein, P-cadherin, significant changes in protein expression are also seen after incubation with SPIONs. In the case of DU 145 cells: a shorter time practically does not change the intensity of the bands, while a longer time slightly increases it [date not shown]. The expression of E-cadherin after treatment of cells with nanoparticles is significantly different depending on the cell line. LNCaP cells, after 24 hours of treatment with SPION/CCh, correspond with a increase in E-cadherin expression, PCNA, but also vimentin. We also observe an soft increase in the expression of the BIM protein, involved - depending on cell type - in cell signaling, in the processes of proliferation and apoptosis. Prolonged treatment of cells does not have a major effect on E-cadherin expression. Normal RWPE1 lineage cells correspond to a marked increase in E-cadherin after 24 h of incubation with SPION/ACh and after 48 h of incubation with SPION/CCh. After 24 h of incubation of DU 145 cells with SPION/CCh, does not significantly affect the expression of E-cadherin but for SPION/CCh an decrease in the concentration of E-cadherin is observed. After longer treatment of the cells, the intensity of the bands is slightly higher than the control sample, and additionally higher intensities were observed for SPION/ACh. Similarly, to DU 145 cells, in LNCaP cell, a higher intensity of bands can be noticed for anionic nanoparticles (after 48 h) compared to cationic ones. Spectacular changes in the expression of E-cadherin are observed for the 22Rv1 line showing the pressure of both E- and N cadherin, and the effect is particularly significant after a shorter incubation time with SPION/CCh. A rather ambiguous effect in Vimentin expression was observed for different prostate cell lines. A decrease in Vimentin expression was observed for PC-3, RWPE and Du145 lines. It should be emphasized that for the Du145 line, the decrease in Vimentin expression is significant. For PC-3 cells, a slight decrease in band intensity was observed after 24 h of incubation, and a slight decrease after 48 h. For the LNCaP and 22Rv1 lines, we observe an increase in the intensity of the SPION/CCh bands and only 10µg/mL SPION/CCh results in a decrease in Vimentin expression for 22Rv1. Comparing all the results, it can be seen that there is no clear relationship between the change in the intensity of the bands and the concentration, type of coating of nanoparticles or incubation time, however, a positive correlated effect of changes in cadherin compression is observed. A similar situation takes place for the ZEB-1 protein: no significant changes in the intensity of the bands are visible and no clear relationship can be found between the incubated cells and the type of nanoparticles. In the case of PC-3 cells, after 24 h of incubation, a slight increase in the intensity of the bands dominates, while after a longer time, a clear decrease in their intensity is visible. For DU 145 cells incubated for both times, there was primarily a slight increase in protein expression. In the case of LNCaP cells, the bands after 48 h of incubation were too faint to be detected. In contrast, this cell line showed clear differences in expression after treatment with SPION/CCh and SPION/ACh. For the first type of nanoparticles, slightly more intense bands were obtained, while for the second - a clear decrease in intensity (after treatment with 10 µg/mL SPION/ACh, a nearly 10-fold decrease was observed compared to the control sample) [date not shown]. For the next protein tested, PCNA, there is no significant change in expression in general. In the case of the PC-3 cell lines, after 24 h of incubation with SPION/CCh and SPION/ACh, a slight decrease in band intensity can be observed, while after the second time - a slight increase or decrease, depends on concentration and type of the SPIONs. For DU 145: all tested concentrations at both times resulted in a decrease in band intensity relative to the control sample. The opposite relationship is visible for LNCaP cells - with the omission of SPION/ACh after 24 h, where there was a decrease in the intensity of the bands. For RWPE1 only higher concentration 10 μg/mL SPION/ACh causes a slight decrease in PCNA expression. Proteins regulating the processes of cell proliferation and apoptosis such as BAX, BIM, BCL-2 were also used in the research. For RWPE, an increase in the concentration of pro-apoptotic protein was observed (with the exception of 10 μg/mL SPION/CCh) with a simultaneous decrease in the concentration of BCL-2 anti-apoptotic proteins. A similar trend was observed for PC-3 cells. Studies involving Akt in cancer cell metabolism are also important. Activation of Akt contributes to a change in the metabolism of the cancer cell. In our studies, we observe a decrease in the expression of this protein for most cells treated with SPIONs especially after a longer incubation. In conclusion, slight changes in protein expression correlate with the results presented earlier, and thus confirm that SPION/CCh and SPION/ACh nanoparticles, in non-toxic concentrations, can be used as, for example, carriers of active substances for prostate cancer cells.

## Discussion

Prostate cancer is one of the most common cancers in men with a fatal outcome but its causes are still not fully understood.^50^ Additional research is therefore needed to find a more precise method of drug delivery, and nanotechnology, in particular its nanoparticle branch, offers new solutions. The efficiency of nanoparticle - based drug delivery is related to the surface modification of specific nanoparticles. Superparamagnetic iron oxide nanoparticles are here a promising tool, which can be used as a complementary therapeutic agent in conventional cancer therapies.^51^ Various research groups,^52^,^53^ have extensively investigated iron-core based nanoparticles, but few applications have been approved for clinical use so far. The main issues limiting their clinical application include low efficacy/degree of drug binding, inadequate tissue selectivity, and deficient SPIONs biodistribution regulation. The effective synthesis of SPION/CCh was performed based on the previously developed methodology^44^ by co-precipitating iron salts in the presence of the cationic derivative of chitosan, which provided them with the colloidal stability in aqueous media. To study the influence of the nanoparticles’ charge (positive or negative) we have additionally introduced a second layer of the anionic derivative of chitosan on the surface of SPION/CCh, obtaining SPION/ACh.^45^ The double-layer coating was created due to the electrostatic interactions between the CCh polycation and ACh polyanion. The fact that both layers of the coating were based on the chitosan backbone allowed us to study the sole influence of the surface charge, without the possible variability introduced by the change in the polymer used. The successful introduction of both layers of the coating was confirmed by the presence of the bands characteristic for trimethylammonium group for SPION/CCh and carboxylic and sulphonic groups for ACh in the respective ATR-FTIR spectra. The effective coating of SPION/CCh with ACh derivative was further supported by the reversal of the charge reflected by the change in the zeta potential value of SPIONs (from + 43.7 mV for SPION/CCh to – 48.9 mV for SPION/ACh), as well as the increased size of the nanoparticles after the second layer was introduced. Even though the colloidal stability of both types of SPIONs in water was high, in the culture medium it was challenged by the high concentration of salts and proteins. As a result, the stability of SPIONs in RPMI-1640 was limited to about 5–6 h, which was further confirmed by the microscopic studies where, in higher concentrations of the nanoparticles, their aggregates were visible after 24 h. SPION/ACh showed higher aggregation tendency. XRD studies confirmed that magnetic cores were small (ca 10 nm) and consisted of maghemite – the oxidized form of magnetite.

Superparamagnetic iron oxide nanoparticles are found to be a promising candidate for use in cancer diagnostics and therapeutic applications. SPIONs are used in cancer treatment using^25^ hyperthermia,^26^ immunotherapy,^54^ drug delivery,^55^ as well as magnetic resonance imaging.^56^ Our research using nanoparticles in a biological environment - in cell culture - allowed us to examine both their properties and their impact on the behavior of cells, as well as to specify their possible impact on cell signaling. Five prostate cancer cell lines were used as a research model, differing in phenotype and, above all, differing in the expression of proteins involved in the EMT transition.

Both SPION/CCh and SPION/ACh demonstrated colloidal stability under all tested conditions, including water, PBS, and cell culture medium (RPMI-1640), at both room temperature and 37 °C, for a 48-hour period. Although slight variations in optical density (OD) were observed depending on the medium and temperature, the values remained low and did not indicate significant aggregation or sedimentation.

SPION/ACh exhibited slightly higher stability in aqueous solutions, while SPION/CCh showed more pronounced stability in PBS and culture medium. This stable dispersion of nanoparticles under physiological conditions supports their suitability for further biological testing, minimizing the impact of nanoparticle aggregation on cytotoxicity and migration assay outcomes.

EMT appears to be a key regulator of cell invasion and metastasis in cancers. Besides the acquisition of migratory/invasive abilities, the EMT process is tightly connected with the formation of cancer stem cells (CSCs), thus contributing to chemoresistance. EMT is then an important therapeutic target for cancer therapies, but its use in clinical practice is still minimal due to, among other things, the heterogeneity of the tumor stadium or the lack of the appropriate drug delivery system. Thus, the attention of researchers is directed to nanomaterials that can be used to counteract the induction of EMT, toward the search for new therapeutic tools in various cancers, including prostate. In order to inhibit the EMT process in cancer cells nanomaterials can be used to modulate several key characteristics, such as migratory/invasive abilities, stem cell phenotype, and tumor progression. This approach was implemented on breast,^57^ colorectal,^58^ melanoma, 59 glioblastoma,^60^ ovarian,^61^ lung or prostate cancer,^62^,^63^ by induction of ROS, decreasing mitochondrial membrane potential, induction of cell cycle arrest and apoptosis, as well as by inhibition of EMT by decreased levels of N-Cadherin, Vimentin, Snail 1 or MMP2. It is consistent with the results we have presented for first time our modified SPION nanoparticles (SPION/CCh and SPION/ACh), for which we observed a decrease in tumor cell migration, an increase in E-cadherin expression, or a decrease in proliferation expression.

## Conclusion

The presented studies conducted on prostate cell lines, using modified SPION nanoparticles, indicate a lack of a clear effect of SPIONs on cell signaling involving EMT proteins. The few changes in E-cadherin expression observed for both the LNCaP and DU 145 lines suggest a more protective effect than a negative one and thus confirm that SPION/CCh and SPION/ACh nanoparticles, in concentrations of 10 µg/mL, can be used as carriers of active substances to prostate cancer cells. This research indicates also the possibility of using SPIONs to capture circulating tumor cells in a biological system.

## Abbreviations

PCTCs, prostate cancer circulating tumor cells; SPIONs, superparamagnetic iron oxide nanoparticles; CTCs, circulating tumor cells; CCh, cationic chitosan; ACh, anionic chitosan; SPION/CCh, SPIONs stabilized with cationic chitosan; SPION/ACh, SPIONs stabilized with cationic and anionic chitosan bilayer; DLS, dynamic light scattering; ELS, electrophoretic light scattering; EMT, epithelial-mesenchymal transition; DDSs, drug delivery systems; MRI, magnetic resonance imaging; PdI, polydispersity index; ZFC, zero field cooled; FC, field cooled; CSCs, cancer stem cells; XRD, X-ray diffraction; ATR, Attenuated Total Reflectance; FTIR, Fourier-Transform InfraRed spectroscopy; OD, optical density; T_b, blocking temperature; SD, standard deviation, EPR, enhanced permeability and retention.
